# The International development of PROQOL-HCV: An instrument to assess the health-related quality of life of patients treated for Hepatitis C virus

**DOI:** 10.1186/s12879-016-1771-0

**Published:** 2016-08-23

**Authors:** Andrew Richard Armstrong, Susan Elizabeth Herrmann, Olivier Chassany, Christophe Lalanne, Mariliza Henrique Da Silva, Eliana Galano, Patrizia M. Carrieri, Vincent Estellon, Philippe Sogni, Martin Duracinsky

**Affiliations:** 1 EA 7334 REMES, Patient-Centered Outcomes Research, University Paris-Diderot, Sorbonne Paris Cité, Paris, France; 2 Australian Institute of Family Studies, Melbourne, Australia; 3 Institute for Immunology and Infectious Diseases, Murdoch University, Murdoch, Western Australia Australia; 4 URC-ECO, Hopital Hotel-Dieu, AP-HP, Paris, France; 5 Centro de Referência e Treinamento DST/Aids, Rue santa Cruz, Sao Paulo, Brazil; 6 U912 (SE4S), INSERM, Marseille, France; 7 Université Paris Descartes, Paris, France; 8 Service d’Hépatologie, Hopital Cochin, AP-HP, Paris, France; 9 Service de Médecine Interne et d’Immunologie Clinique, Hopital Bicetre, AP-HP, Kremlin-Bicetre, France

**Keywords:** Questionnaires, Quality of life, Hepatitis c, Health status, Research methods

## Abstract

**Background:**

Hepatitis C virus (HCV) compromises Health-related Quality of Life (HRQL) with detriments to Physical, Mental and Social health domains. Treatment with interferon and ribavirin is associated with side effects which further impair HRQL. New treatments appear potent, effective and tolerable. However, Patient Reported Outcomes instruments that capture the impact on HRQL for people with hepatitis C are largely non-specific and will be needed in the new treatment era. Therefore, we developed a conceptually valid multidimensional model of HCV-specific quality of life and pilot survey instrument, the Patient Reported Outcome Quality of Life survey for HCV (PROQOL-HCV).

**Methods:**

HCV patients from France (*n* = 30), Brazil (*n* = 20) and Australia (*n* = 20) were interviewed to investigate HCV-HRQL issues raised in the scientific literature and by treatment specialists. Interviews were recorded, transcribed and translated into English and French.

**Results:**

Fifteen content dimensions were derived from the qualitative analysis, refined and fitted to four domains: (1) Physical Health included: fatigue, pain, sleep, sexual impairment and physical activity; (2) Mental Health: psychological distress, psychosocial impact, and cognition; (3) Social Health: support, stigma, social activity, substance use; (4) Treatment: management, side effects, and fear of treatment failure. The impact of some dimensions extended beyond their primary domain including: physical activity, cognition, sleep, sexual impairment, and the three treatment dimensions. A bank of 300 items was constructed to reflect patient reports and, following expert review, reduced to a 72-item pilot questionnaire.

**Conclusion:**

We present a conceptually valid multidimensional model of HCV-specific quality of life and the pilot survey instrument, PROQOL-HCV. The model is widely inclusive of the experience of hepatitis C and the first to include the treatment dimension.

## Background

Chronic infection with the hepatitis C virus (HCV) results in morbidity and mortality related to hepatic and extra-hepatic disease processes. An estimated 130 and 170 million people worldwide are HCV-seropositive. The blood-borne virus is transmitted by unsafe medical and drug injecting practices and sexually when mucosal integrity is breached [1]. In specific contexts, such as prisons [2], regions with high prevalence and insufficient prevention measures [1], and in HIV-seropositive men [3], the risk of transmission is increased.

Anti-viral treatment can eradicate the virus, improve hepatic histology and prevent liver-related death but complications of chronic infection and toxicity associated with treatment can impact on health-related quality of life (HRQL) by diminishing physical, emotional and social functioning [4]. Second generation agents based on a triple combination therapy (PEG-IFN + ribavirin + a protease inhibitor: boceprevir or telaprevir) increased HCV clearance rates but augmented toxicity and decreased HRQL [5]. However, enhanced HRQL through symptom alleviation, economic and social benefits, for example, work force participation and removal of social stigma, can follow after successful treatment [6].

New drugs that target the virus directly are available or in clinical trial phase. These treatments, given without concomitant interferon alpha, can result in sustained virological response (SVR) in over 90 % of cases. Notably, toxicity is reduced in comparison with second generation triple combinations, although response remains influenced by genotype, stage of fibrosis and pre-existing drug-resistant mutations [7, 8]. Unfortunately, the price of such drugs limits their accessibility and many people are waiting to be treated [9]. Regardless, new therapies raise the possibility of eradicating hepatitis C and reducing morbidity and mortality [10].

Patient-Reported Outcome (PRO) measures are important to evaluate the impact of chronic infection; eligibility and readiness for treatment; and assessment of HRQL during and post treatment. These measures ensure patient experiences and preferences are captured in order to discriminate between treatment options [10]. Since PRO measures provide insight into the patient’s perspective there must be evidence that the measures reflect that perspective, this is content validity. A three-tiered literature review of 74 articles by Kleinman and colleagues [11], found 22 qualitative studies that identified chronic HCV-related concepts from which they selected five on the basis that they were evidently important to patients, easily implemented into a clinical trial and had potential for sensitivity to change. These five concepts were: depression/anxiety, fatigue, flu-like symptoms, cognitive function, and insomnia [11]. Furthermore the literature suggested that sexual function [12], stigma [13], co-infection [14], resilience [15], emotional volatility [16] and treatment-related impacts [17] were important unmeasured features. In the next step, they examined what PRO measures had been used in clinical trials and found that these instruments provided inadequate coverage of the concepts; and finally, that of 18 existing measures only four were validated in PRO populations and only one demonstrated content validity in the chronic HCV population, the Hepatitis Quality of Life Questionnaire (HQLQ) (13).

Until recently [18, 19], there was no survey instrument measuring, comprehensively, the HRQL of HCV patients or included a treatment dimension. Generic measures such as the MOS Health Survey Short-Form 36 (SF-36), followed by the Hepatitis Quality of Life Questionnaire (HQLQ) [20] which contributes two short HCV-specific subscales to the SF-36 are often employed. Other HRQL instruments used in chronic liver disease, of which HCV is a leading cause are: The Chronic Liver Disease Questionnaire (CLDQ) [21], the Liver Disease Quality of Life Questionnaire (LDQOL) [22], and the Liver Disease Symptom Index 2.0 (LDSI) [23].

Ideally, HRQL instruments should be applicable across diverse populations but most instruments were developed in single contexts and translated into target languages when required. These instruments are not robust to semantic differences between languages or cultural perceptions of health. A validated questionnaire sensitive to these factors should offer precision in terms of understanding patient perspectives, monitoring clinical impacts during treatment, and generating uniform clinical trial data.

### Aims

**Fig. 1:**
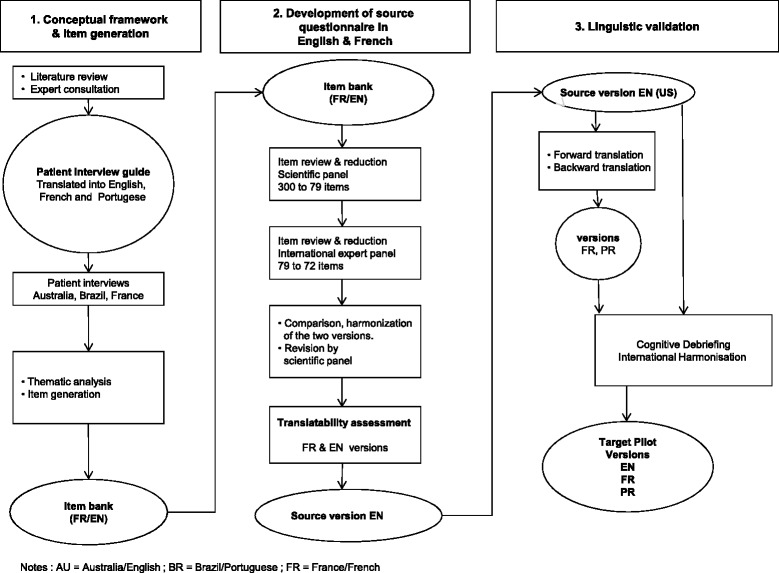
PROQOL HCV Development process AU = Australia/English ; BR = Brazil/Portuguese ; FR = France/French

We aimed to develop a contemporary HCV-specific, multidimensional, HRQL survey instrument, sensitive to treatment, in three representative languages: English, French and Portuguese. The development of PROQOL-HCV was concordant with current guidelines [24, 25] and involved a four stage process (Fig. 1): (1) development of a conceptual model and item generation; (2) development of a pilot questionnaire; (3) linguistic and (4) psychometric validation. Three stages are presented here and the psychometric validation in a second manuscript. The research was conducted in Australia, Brazil, and France.

## Methods

### Specialist consultation

Ten experts contributed knowledge concerning current issues relevant to HCV HRQL to build the conceptual model; a scientific panel (physician, psychologist, nurse, patient advocate) involved in hepatitis C management and research conducted the qualitative analysis of interviews, the item generation and subsequent reduction. An international expert panel participated in the second step of item reduction. Professional translators were used to achieve harmonisation between the three languages.

### Patients

Seventy patients with HCV were recruited for interview from hospital outpatient clinics in: Australia (AU), Brazil (BR) and France (FR). Patients were selected to reflect the diversity of the general epidemic; were over the age of 18 years and able to provide written informed consent. The study protocol conformed to the ethical guidelines of the 1975 Declaration of Helsinki and was approved by local Institutional Review Boards: CPP Ile de France 4, Paris, France; the Royal Perth Hospital (EC2010/103) and Murdoch University (EC2011/002) Western Australia; and the STD/AIDS Reference and Training Center, State in San Paulo (EC2010/010).

### Stage 1: Conceptual model

The first stage defined the conceptual model of HCV HRQL and generated items to reflect this model. Themes reflecting the lived experience of HCV were established through literature review, specialist consultation, and particularly patient interviews. From patient interview syntax a bank of potential items for a pilot PROQOL instrument was extracted.

#### Patient interview guide

Medline was interrogated across the period of 1990 – 2011 to identify HCV HRQL-relevant qualitative and quantitative research. HCV HRQL issues highlighted in qualitative research were cross-referenced with the content of instruments used in quantitative research to measure HCV HRQL to identify which issues were measured and which were not. Ten HCV experts were likewise consulted to identify issues not covered by existing instruments. An interview guide was subsequently developed to explore existing and newly identified themes. The guide comprised six open-ended questions concerning the impact of HCV on activities of daily life and seventy-three semi-structured questions spread across the topic areas: physical, psychological and social health; stigma; self-image and self-esteem; resilience; and treatment.

#### Patient interviews

A total of seventy semi-directed interviews were conducted with the patient interview guide. These were performed, face to face, in English, Portuguese and French, respectively, in 2011 (*n* = 20 in AU; *n* = 20 in BR; and 30 in FR) by experienced interviewers. A further five, in France, explored to a greater extent the impact of new therapies. In establishing sample size, we erred on the side of safety and aimed for at least 20 interviews per country to ensure thematic saturation [26]. France included more participants to ensure inclusion of people on the newer drugs that were at the time unavailable outside of clinical trials in Brazil and Australia. Interviews of 1–2 h duration were recorded and transcribed in the original languages.

Sample sociodemographic and clinical characteristics are shown in Table 1. Information collected included age, gender, ethnicity, employment status and family living arrangements; and relevant health history, including HCV biomarkers, symptoms and treatment side effects measured by a self-report questionnaire developed by Justice and colleagues [27] and adapted by the French National Agency for Research on HIV/AIDS and viral hepatitis (ANRS). The software package R (www.r-project.org/) was employed to generate descriptive statistics.

**Table 1:** Participant characteristics by country

Variables	Australia *n* = 20	Brazil *n* = 20	France *n* = 30	Total *N* = 70
Demographic factors				
Male Sex	17 (85.0)	6 (30.0)	21 (70.0)	44 (62.9)
Age	48.7 (6.0)	51.8 (9.4)	50.45 (9.7)	50.3 (8.5)
Weight (kg)	83.0 (19.9)	66.8 (15.4)	70.5 (13.4)	73.0 (17.1)
Married/partner	5 (25.0)	11 (55.0)	17 (56.7)	33 (47.1)
Ethnicity Caucasian	18 (90.0)	12 (60.0)	23 (82.1)	53 (75.7)
Living alone	7 (35.0)	2 (10.0)	9 (30.0)	18 (25.7)
Living with children	4 (20)	10 (50.0)	14 (46.6)	25 (35.7)
Education ≥ university	3 (15.0)	4 (20.0)	7 (23.3)	14 (20.0)
Employment^a^	12 (60.0)	6 (30.0)	20 (68.9)	48 (69.6)
Current smoker	12 (60.0)	7 (35.0)	15 (50.0)	34 (48.6)
Alcohol last month	15 (75.0)	5 (25.0)	13 (44.8)	33 (47.8)
Past history of Injecting drugs	17 (85.0)	3 (15.8)	14 (48.3)	34 (50.0)
Treatment^b^				
Past history	5 (26.3)	2 (10.0)	17 (58.6)	24 (35.3)
Current	14 (70.0)	15 (75.0)	13^c^ (46.4)	42 (61.8)
Pill burden (total number)	8.1 (6.3)	8.2 (4.8)	9.0 (5.4)	8.5 (5.4)
Antidepressant drugs	7 (35.0)	12 (60.0)	5 (16.7)	24 (34.3)
Antianxiety drugs	5 (20.0)	4 (20.0)	4 (13.3)	13 (18.6)
Sedative drugs	4 (20.0)	0 (0.0)	5 (17.9)	9 (13.4)
Clinical factors				
Time since diagnosis (years)	5.0 (13.8)	3.5 (4.5)	8.0 (10.3)	5.0 (10.0)
HIV-Coinfection	5 (29.4)	5 (27.8)	4 (26.8)	14 (28.0)
Depression, self-reported (past month)	11 (55.0)	10 (50.0)	12 (41.4)	33 (47.8)
Symptoms (total number)	12.0 (7.0)	15.0 (13.5)	12.5 (6.0)	13.0 (7.0)

^a^Inclusive of full time and part time work; ^b^Both interferon alpha and pegylated in combination with ribavirin; ^c^8 patients (32 %) receiving directly acting antiviral drugs

Data are summarised as count (percentage) for categorical variables, and mean (SD) for numerical variables

#### Thematic analysis

Transcripts were translated into English and French by professionals. The analysis of 70 interviews was performed by the scientific panel and a qualitative approach was taken to identify recurring themes, commonalities and variations until data saturation was reached at 29 themes. Two pairs of Anglophone and Francophone investigators coded approximately 18,000 lines of syntax to one or more (when concepts overlapped) emerging themes. Differences in coding were discussed until consensus was reached. Syntax were subsequently sorted and pooled by code. Theme content was examined for distinctiveness, cross-over and redundancy. The 29 grounded and frequently overlapping themes were refined and parcelled into 15 broad and distinct dimensions.

#### Item generation

Raw statements that exemplified the content of each dimension were identified and converted into grammatically and syntactically correct items to form a bank of 300 potential survey items in Excel, in French and English. Each item is written with the expectation that a respondent will indicate the extent to which it applies to them on five point Likert scale (0 = “always”, 1 = “often”, 2 = “from time to time”, 3 = “rarely”, 4 = “never”).

### Stage 2: Development of the pilot instrument

The goal was to reduce the number of items contained in the item bank to a smaller pool representative of the HCV HRQL dimensions identified during the interview process. Each item was reviewed on ten criteria using a three-point scale (1 = Yes, 2 = No, 3 = Unsure): (1) Item content inconsistent with the theme, dimension and domain definitions (Item relevance); (2) Item similar in wording (semantics) to other items (Item redundancy); (3) Item measures general HRQL rather than HCV HRQL (Item disease-specific); (4) Item confusing rather than clear (Item clarity); (5) Item complex rather than simple (Item cognitive burden); (6) Item translatability issue; (7) Item cross-cultural fit issue; (8) Item does not fit with a designated response category (frequency, intensity, quantity); (9) Item not sensitive to change (Item sensitivity); (10) Item less explicit than another existing item. Seventy-nine items were retained on majority agreement. These 79 items were passed to the international expert panel, who used a three-point scale (1 = Yes, 2 = No, 3 = Unsure) to review each item on four criteria: (1) relevance to theme, dimension and domain definitions; (2) item clarity and comprehensibility; (3) sensitivity to change; (4) and retain or delete item. Seventy-two of the 79 items were retained on majority agreement to form the pilot instrument.

It is expected that psychometric analysis and validation of the pilot instrument will result in subscale item groupings based on shared content, and will reduce the number of items to the minimum necessary to reflect our conceptual model and adequately measure HCV HRQL. Subscale scores based on the sum of raw item scores will be rescaled to range from 0 to 100 for ease of comparison.

### Stage 3: Linguistic validation

English and French versions of the pilot questionnaire underwent translatability assessment by two linguists specialized in the development of PRO instruments. Several items were reformulated and content specified. Forward translation into French and Portuguese was carried out and back-translated into English. Three draft questionnaires, in English, French and Portuguese were compared and harmonised. Discrepancies were resolved and cognitive debriefing was performed on a new 20 patient sample to check concept interpretation.

## Results

The results describe the participant characteristics; themes that emerged from participant interviews, and the conceptual model that informed selection of items for inclusion in the PROQOL-HCV pilot questionnaire.

### Participant characteristics

**Fig. 2:**
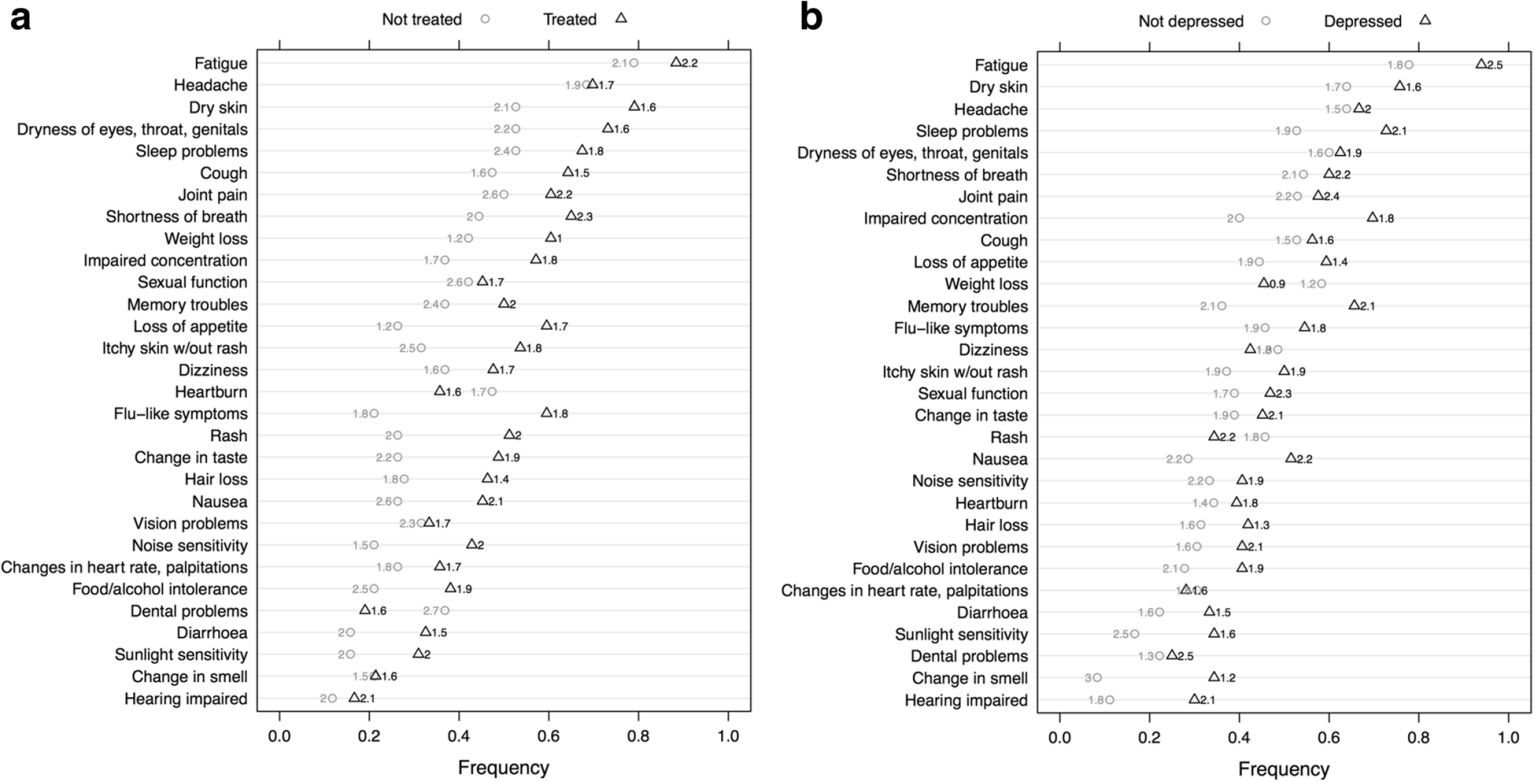
**a** Symptoms by current HCV antiviral treatment status: Not treated O ; Treated Δ. Symptom discomfort range 0 = Not at all to 3 = Very discomforting. **b** Symptoms by self-reported depression status: Not depressed O ; Depressed Δ. Symptom discomfort range 0 = Not at all to 3 = Very discomforting

Socio-demographic and clinical characteristics of the 70 interviewees are shown in Table 1. Participants (mean age = 50 years) were predominantly male in Australia and France, and female in Brazil. Risk exposure to hepatitis C varied between countries. The majority of Australians and half the French reported a history of injecting drug use (IDU) in comparison with three Brazilian patients. Fourteen patients were co-infected with HIV. Hepatitis C virus genotype 1 was prevalent in over 60 % of cases (information on 53 patients). French patients had been diagnosed earlier than the other groups but this was not thought to necessarily indicate a longer duration of infection. In France and Brazil approximately half of participants were living with a partner and children in contrast with Australians who more commonly lived alone. The French and Australians were more likely to be employed, although 40–70 % of participants reported not working at all. According to the measure used to assess perceptions of social status [28] most of the participants ranked themselves just less than half way between people of lowest and highest status in society. Smoking and alcohol rates were higher in Australians. Participants (62 %) were receiving antiviral drugs, mostly combination interferon and ribavirin, and 8 French patients were treated with directly acting antivirals. Five additional interviews (not presented in Table 1) were conducted with French patients on IFN-free regimens. Ten patients were receiving treatment for both depression and anxiety (15 %). The most frequently reported symptoms were: fatigue, headache and dry skin. Those on treatment were more likely to complain of flu-like symptoms and loss of appetite (chi square test; *p* = 0.012; *p* = 0.034) and people reporting depression (*n* = 33) were more likely to complain of impaired memory and concentration; and changes in olfactory perception (*p* = 0.029; *p* = 0.027, *p* = 0.019). Symptom discomfort (rated 0 to 3) was higher for the untreated group for dental (*p* = .032) and sleep problems (*p* = .025), and higher for people reporting depression for fatigue (*p* = .003) and dental problems (*p* = .017) but lower for changes in olfactory perception (*p* = .002) (Fig. 2a-b).

### Thematic analysis

**Fig. 3:**
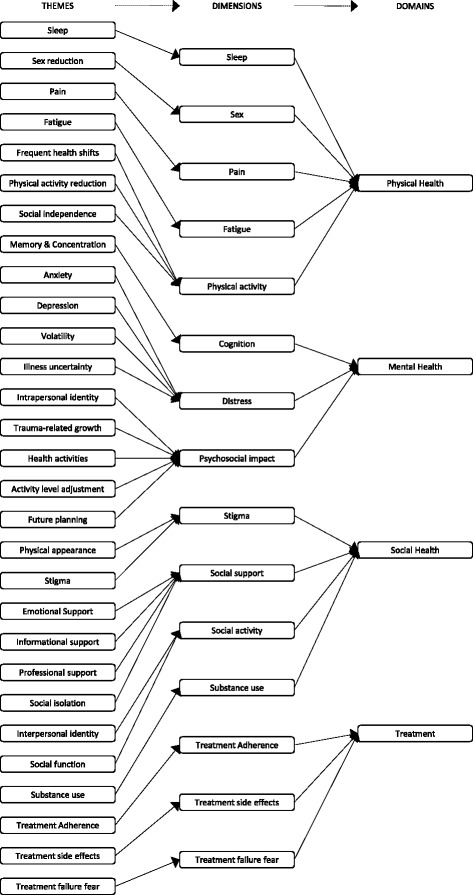
Conceptual model of HCV-specific health-related quality of life

Analysis of interview data revealed 29 themes constituting hepatitis C-specific health-related quality of life in the context of both chronic infection and treatment conditions. These were parcelled into 15 dimensions representing the three overarching domains of Physical Health, Mental Health, Social Health, and a fourth domain, HCV Treatment, that is new to this study. These themes, dimensions and domains constitute our conceptual model of HCV HRQL (Fig. 3), and accord with prevailing HRQL theory [29].

Physical Health incorporated fatigue, pain, sleep and sexual impairment and physical activity; Mental Health included psychological distress, psychosocial impact, and cognition; Social Health incorporated support, activity, stigma, and substance use. The impact of some dimensions extended beyond their primary domains to affect others including sleep and sexual function, concentration, memory and treatment burden. The fourth domain assigned as ‘Treatment’ had influence on aspects of the three other domains with respect to treatment adherence, side effects, and treatment failure fears.

1. **Physical Health:** The pain dimension incorporated themes of pain intensity and frequency and the extent it interfered with physical, mental and social activities. Pain was manifested by musculoskeletal discomfort, morning stiffness, and ‘all over pain’. Fatigue was framed by disrupted physical, mental and social activity. Participants described chronic low to medium intensity fatigue with debilitating, sporadic high intensity episodes typically occurring after injection of interferon. Physical activity was constrained by health fluctuations that disrupted routines and planned activities. The need for rest limited mobility and travel distances and resulted in a greater reliance on others, especially family members, to carry out daily tasks. It was perceived that partners underappreciated the intensity of fatigue. A waning capacity for activity was accompanied by a diminution of interests. The sleep dimension incorporated difficulties falling or remaining asleep, and disturbances arising from anxiety, pain and itching which effected alertness the next day. Depressive and treatment-related symptoms and contagion/transmission anxieties resulted in negative impacts on sexuality, including reduced libido, difficulty maintaining arousal and achieving orgasm.
2. **Mental Health***:* Depression, anxiety, stress and struggles associated with antiviral treatment resulted in distress. Participants described shock, disbelief, grief, frustration, and helplessness. Feelings of nervousness, heightened irritability, anger and rage were disturbing and unexpected. Participants were concerned their infections might be incurable and felt a loss of control and independence. Some viewed their futures to be bleak and in some cases were fearful of dying – especially those who were parents; and emotional distress disrupted relationships.
  Positive and negative psychosocial impacts reflecting adjustments to HCV-associated emotional reactions were described. Mental health-related changes resulted in an altered self-perception and expression of personality. Appreciation of life was re-appraised and goals adjusted, and some described a greater respect and empathy for others. Behavioural changes in health-seeking practices were related to diet, exercise, usage of complementary therapies and altered hygiene routines. Cognitive and behavioural strategies included: positive thinking, for example, that side effects of medication were part of the healing process, determination to complete the treatment course, meditation and prayer. Problems with concentration and memory interfered with maintaining schedules, locating essential tools and completing sequential steps in a task. Cognition was disrupted by sleep disturbances and fatigue.
3. **Social Health:** Encompassed the sense of being cared for by family and friends; and professional and informational support. Some participants described losing social invitations, companionship and marital/relational instability but others described feeling cared for and valued. Regular engagement with health professionals supported confidence, sense of control and medication adherence. Loss of social activity drew together, thematically, the interest, accessibility, capacity and satisfaction with typical daily social activity following a diagnosis of HCV. Opportunities to participate in group sporting activities due to infectivity, and avoidance of drugs and alcohol, limited social interaction as did limited energy and a sense of lost attractiveness. HCV-related stigma infused the Social Health domain and influenced personal and social identity. Self was perceived as ‘diseased’ and there was a fear of disclosing HCV sero-status and transmitting the infection. In addition, discrimination in healthcare settings, the workplace, and from family and friends was evidenced. Stigma concerns often resulted in socially avoidant behaviour.
  The use of substances was characterised by positive and negative statements related to coping with reduced substance use leading to better health but also the loss of socialisation with friends connected through the use of drugs and alcohol. Some failed to cope with the abstinence required for treatment eligibility and struggled to overcome substance dependency.
4. **Treatment:** Treatment impacted the Physical, Mental and Social domains of HRQL in complex ways. Participants undergoing hepatitis treatment were required to make life adjustments for the duration of therapy. Regimen adherence was described as a ‘responsibility’ which restricted travel and required concealment. Prior to treatment there was fear of side effects and treatment inefficacy. Side-effects including changes in appearance like weight loss, jaundice and hair loss affected self-esteem and sense of social acceptability sometimes resulting in treatment cessation (or interruption).
  Other participants felt ready to cope and some with the means to do so, made plans to withdraw socially and/or from employment to complete treatment. Determination was evident with hopes of longevity promoting persistence with treatment, others were anxious about treatment failure or recurrent infection. Side effects that impacted mood were troublesome, including aggression and irritability, sexual function, personal appearance and interruptions to travel plans. In stark contrast with people taking interferon, those treated with DAAs reported feelings of cheerfulness, approaching euphoria, and were reluctant to stop their treatment.

### Thematic differences based on country of origin

While the interview data mostly revealed commonalities in health-related quality of life matters between participants from the three countries, some differences emerged. Brazilian interviewees were more likely to identify their health as concerning their families. They were more likely to live in shared spaces with family and non-family members and express concern around interpersonal hygiene. In contrast participants from French and Australian cultures were more likely to identify their health as a self-concern rather than a family concern and more often lived alone or in couples. French interviewees emphasised their fears for the future more than other participants. Australian interviewees commonly reported ceasing employment and reducing their level of social engagement for the period of treatment whereas participants from Brazil and France did not. Practicing religion and prayer helped many of the Brazilian interviewees to cope whereas other participants very rarely raised this.

## Discussion

We developed a conceptual model and pilot survey instrument of HCV-specific HRQL in-line with US Federal Drug Administrion (FDA), and European Medicines Agency (EMA) guidelines [25, 30]. The model and instrument incorporate three broad HRQL domains of Physical, Mental and Social Health, and a fourth – Treatment - that captures patient experience with interferon-based and interferon free regimens. The model and pilot instrument embody the breadth of issues impacting the quality of life of people affected by hepatitis C and clearly demonstrate content validity. Other HRQL instruments identify and elucidate one or more of these themes, but do not unify them within a comprehensive HCV-specific HRQL model. This may reflect of a lack of heterogeneity in the samples during the instruments’ development which undermines their content validity.

In contrast we collated an item bank from a qualitative sample reflecting demographic diversity in terms of disease stage, treatment status, sex, linguistic, educational and healthcare contexts. Furthermore, subsumed within our dimensions, are disease-nuanced items tapping important issues not incorporated in other instruments. For instance, while distress is a common element of HRQL measurement, our model identifies HCV-specific distress arising from: contagiousness concerns, guilt and worry about impact on family, fear of complications, uncertainty, around the trajectory of the illness, emotional volatility and irritability. Likewise, subjectively experienced deficits in attention, concentration and memory that impact on quality of life are included and can provide additional information to objective performance tests. Our instrument incorporates sexual function as does other HRQL instruments, but importantly discriminates dysfunction from reduced sex drive, problems with sustaining arousal, or stigma affecting partner availability. Few HCV-HRQL instruments capture internalised stigma, i.e. a sense of ‘self’ as contagious, in additional to interpersonal fears of social rejection.

We sought to address the cultural nuances that emerged by finding common threads across the Brazilian, French and Australian interviews. We adapted item formulation to retain the same content meanings across the three languages with the intention of strengthening and enhancing the content validity of the items.

Our conceptual model incorporates the impact of treatment into HRQL assessment. This is novel. This instrument was developed in the context of chronic infection, HIV co-infection and changing treatment strategies. Identifying fluctuation or changes in HRQL in response to interventions is critical to effective monitoring of patient outcomes and PROQOL-HCV captures these nuances.

Psychometric assessment, refinement and validation of the pilot PROQOL HCV survey instrument is currently underway. It has been administrated to samples of HCV patients in France, Brazil and Australia. We intend to publish a short version of the survey for use in large multi-focus studies where space is limited, and a long version for use in more focused HCV HRQL investigations, as we have done for HIV HRQL research [31].

We acknowledge limitations. A degree of subjectivity was inevitable in the identification and extraction of interview themes and other researchers may have proposed a different nomenclature for the themes or their dimensional arrangement but the development of the instrument was evidence-based and consensus driven. PROQOL-HCV is a disease-specific instrument and is therefore limited in application to the management of hepatitis C.

The decision to conduct this study in Brazil, France and Australia was to an extent pragmatic since the teams had collaborated successfully in the past and therefore we were confident that the project could be implemented feasibly in these countries. However, there are features of the epidemic and access to treatment that are informative across the regions. Furthermore, previous experience with large international studies had led us to the observation that development of HRQL in a single country is not satisfactory. However including too many countries in the simultaneous development would prolong the development process unnecessarily. Our approach also meant that idiomatic expression and other difficulties of expression and meaning could be resolved during the item generation phase and our pilot instrument would be the more robust because of this. It is the case that linguistic validation/cultural adaptation will be needed for countries not speaking French, English or Portuguese but this method had been used for many other instruments.

## Conclusion

Potent anti-viral alternatives to IFN and ribavirin have improved the possibility of achieving a sustained virological clearance. The arrival of new treatments, with comparably high effectiveness, low toxicity and high prices, call for using disease-sensitive HRQL measures to support decisions regarding treatment choice. Successful treatment limits liver-related morbidity and increases health related quality of life [32]. However, mathematical modelling suggests that HCV is under-diagnosed, people with existing infections are numerous and that better clinical management of infected people is required [33]. Given the evident HRQL impacts of HCV we recommend embedding monitoring of patient-reported outcomes in clinical care. PROQOL-HCV, derived from our HCV-specific conceptual model, shows promise as a comprehensive and discriminating PRO instrument sensitive to the full range of disease and treatment experiences of HCV patients, clearly distinguishing it from existing HRQL measures.

## Abbreviations

AU: Australia
BR: Brazil
CLDQ: Chronic liver disease questionnaire
EMA: European Medicines Agency
FDA: US Federal Drug Administration
FR: France
HCV: Hepatitis C virus
HCV-HRQL: Hepatitis C Virus-specific Health Related Quality of Life
HIV/AIDS: Human Immunodeficiency Virus/Acquired Immune Deficiency Syndrome
HQLQ: Hepatitis quality of life questionnaire
HQLQ: Hepatitis quality of life questionnaire
HRQL: Health-related quality of Life
IDU: Injecting drug use
LDQOL: Liver disease quality of life questionnaire
LDSI: Liver disease symptom index
PEG-IFN: Pegylated interferon
PRO: Patient-reported outcome
PROQOL-HCV: Patient reported outcome quality of life survey for HCV
SF-36: MOS health survey short-form 36
SVR: Sustained virological response
